# NSAID-activated gene 1 mediates pro-inflammatory signaling activation and paclitaxel chemoresistance in type I human epithelial ovarian cancer stem-like cells

**DOI:** 10.18632/oncotarget.12355

**Published:** 2016-09-30

**Authors:** Ki-Hyung Kim, Seong-Hwan Park, Kee Hun Do, Juil Kim, Kyung Un Choi, Yuseok Moon

**Affiliations:** ^1^ Department of Biomedical Sciences, Pusan National University School of Medicine, Yangsan, South Korea; ^2^ Biomedical Research Institute and Pusan Cancer Center, Pusan National University Hospital, Busan, South Korea; ^3^ Department of Obstetrics and Gynecology, Pusan National University School of Medicine, Busan, South Korea; ^4^ Department of Pathology, Pusan National University School of Medicine, Busan, South Korea; ^5^ Research Institute for Basic Sciences, Pusan National University, Busan, South Korea

**Keywords:** NAG-1, epithelial ovary cancer, paclitaxel, NF-κB, chemoresistance

## Abstract

Epithelial ovarian cancer (EOC) remains the most lethal gynecologic malignancy in developed countries. Chronic endogenous sterile pro-inflammatory responses are strongly linked to EOC progression and chemoresistance to anti-cancer therapeutics. In the present study, the activity of epithelial NF-κB, a key pro-inflammatory transcription factor, was enhanced with the progress of EOC. This result was mechanistically linked with an increased expression of NSAID-Activated Gene 1 (NAG-1) in MyD88-positive type I EOC stem-like cells, compared with that in MyD88-negative type II EOC cells. Elevated NAG-1 as a potent biomarker of poor prognosis in the ovarian cancer was positively associated with the levels of NF-κB activation, chemokines and stemness markers in type I EOC cells. In terms of signal transduction, NAG-1-activated SMAD-linked and non-canonical TGFβ-activated kinase 1 (TAK-1)-activated pathways contributed to NF-κB activation and the subsequent induction of some chemokines and cancer stemness markers. In addition to effects on NF-κB-dependent gene regulation, NAG-1 was involved in expression of EGF receptor and subsequent activation of EGF receptor-linked signaling. The present study also provided evidences for links between NAG-1-linked signaling and chemoresistance in ovarian cancer cells. NAG-1 and pro-inflammatory NF-κB were positively associated with resistance to paclitaxel in MyD88-positive type I EOC cells. Mechanistically, this chemoresistance occurred due to enhanced activation of the SMAD-4- and non-SMAD-TAK-1-linked pathways. All of the present data suggested NAG-1 protein as a crucial mediator of EOC progression and resistance to the standard first-line chemotherapy against EOC, particularly in MyD88-positive ovarian cancer stem-like cells.

## INTRODUCTION

Epithelial ovarian cancer (EOC) remains the most lethal gynecologic malignancy and one of the leading causes of cancer-related mortality among women in developed countries [1–3]. Due to the absence of symptoms and lack of effective screening strategies during its early stages, ovarian cancer typically progresses to the advanced stages [4–6]. However, EOC is not a single disease but is composed of a diverse group of tumors that can be classified into type I and type II based on distinctive morphologic and molecular genetic features [56]. Type I consists of low grade tumors (serous, endometroid), mucinous and clear cell and are indolent, less aggressive, genetically stable and devoid of p53 mutations. Type II tumors consist high grade, aggressive and genetically instable tumors with overwhelming p53 mutations. Every step in ovarian carcinogenesis, including tumor initiation, promotion, and progression, is seriously affected by inflammatory responses [7–10]. Chronic ovarian inflammatory diseases including endometriosis have been extensively investigated as potential predisposing etiological factors of EOC. As the central transcription factor regulating pro-inflammatory responses, nuclear factor-κB (NF-κB) modulates the production of cytokines, growth factors, and anti-apoptotic proteins, many of which are strongly linked to cancer progression and chemoresistance to anti-cancer therapeutics [11–14]. Moreover, NF-κB-mediated chemokines can be critical mediators in the tumor microenvironment and contribute to cancer progression [15–20]. Thus, chemokine- or chemokine receptor-expressing ovarian cancers are aggressive with poorer outcomes [21–23]. For instance, prolonged NF-κB activation and the subsequent production of pro-inflammatory chemokines in myeloid differentiation protein 88 (MyD88)-expressing epithelial ovarian cancer cells are involved in chemoresistance to paclitaxel, a crucial drug for standard first-line chemotherapy [24, 25]. Based on their chemo-response, EOC cells are classified into 2 groups: chemoresistant (type I) and chemosensitive (type II) [26, 27]. Type II EOC cells represent classical ovarian cancer cells and are characterized by fast growth and cell division with a lack of cell-to-cell contact inhibition. Type I cells are characterized by slower growth, cancer stem-like properties, and a high level of MyD88 [24], which contributes to ovarian cancer progression by inducing epithelial ovarian cancer cell proliferation [28], survival [15–20], and metastasis [29], as well as tumor angiogenesis [30] and paclitaxel chemoresistance [24]. Moreover, inflammation as a result of injury affects the self-renewal and differentiation of type I EOC cells [27]. Inhibition of inflammation prevents tumor repair and renewal of EOC stem cells and may have a significant effect on disease recurrence.

Epidemiologic studies have shown that non-steroidal anti-inflammatory drugs (NSAIDs) can reduce the risk of various types of cancers including ovarian cancer [12, 31–36]. At the molecular level, aspirin, a traditional NSAID, acetylates the p53 tumor suppressor protein and modulates the expression of p21^CIP1^, an inhibitor of cell cycle progression, as well as Bax, a pro-apoptotic protein [37]. In addition, several lines of evidence indicate that NSAIDs induce the expression of NSAID-activated protein 1 (NAG-1), which facilitates the anti-cancer activity of NSAIDs in many cancer cells [38–41]. NAG-1, also known as macrophage inhibitory cytokine 1 (MIC-1), growth differentiation factor 15 (GDF-15), prostate-derived factor (PDF), placental bone morphogenetic protein (PLAB), and placental transforming growth factor-β (PTGF-β), is a distant member of the transforming growth factor-β (TGF-β) superfamily. Although most normal tissues express low quantities of NAG-1, pathologic conditions including acute injuries, chronic inflammation, and malignancy can induce relatively high levels of epithelial NAG-1 expression [42–48]. Therefore, serum NAG-1 is clinically useful in the diagnosis and prediction of some chronic inflammatory and malignant diseases [49–52]. Particularly, in terms of epithelial carcinogenesis, elevated levels of serum NAG-1 have been directly correlated with the progression of tumors to metastasis [44, 51, 53]. Mechanistically, NAG-1 plays crucial roles in the modulation of metastatic cancer cell survival and motility in the extracellular matrix and circulation [50, 54, 55].

Although chronic pro-inflammatory activation could be of great importance in ovarian cancer cell recurrence and chemoresistance, the underlying mechanism and its roles are not yet fully understood. Here, we assessed NAG-1 as a potent modulator of pro-inflammatory signals in epithelial ovarian carcinoma cells. Cellular signaling responses were assessed using epithelial ovarian cancer cells, particularly focusing on the type I EOC stem-like cells, to evaluate pro-inflammatory chemokine production and chemoresistance. The dissection of NAG-1 biology in type I EOC cells could provide better insight into early diagnostic, therapeutic, and preventive strategies against chronic ovarian inflammation-associated EOC recurrence and chemoresistance.

## RESULTS

### Persistent activation of NF-κB signals and enhanced induction of chemokines in human ovarian cancer

**Figure 1:**
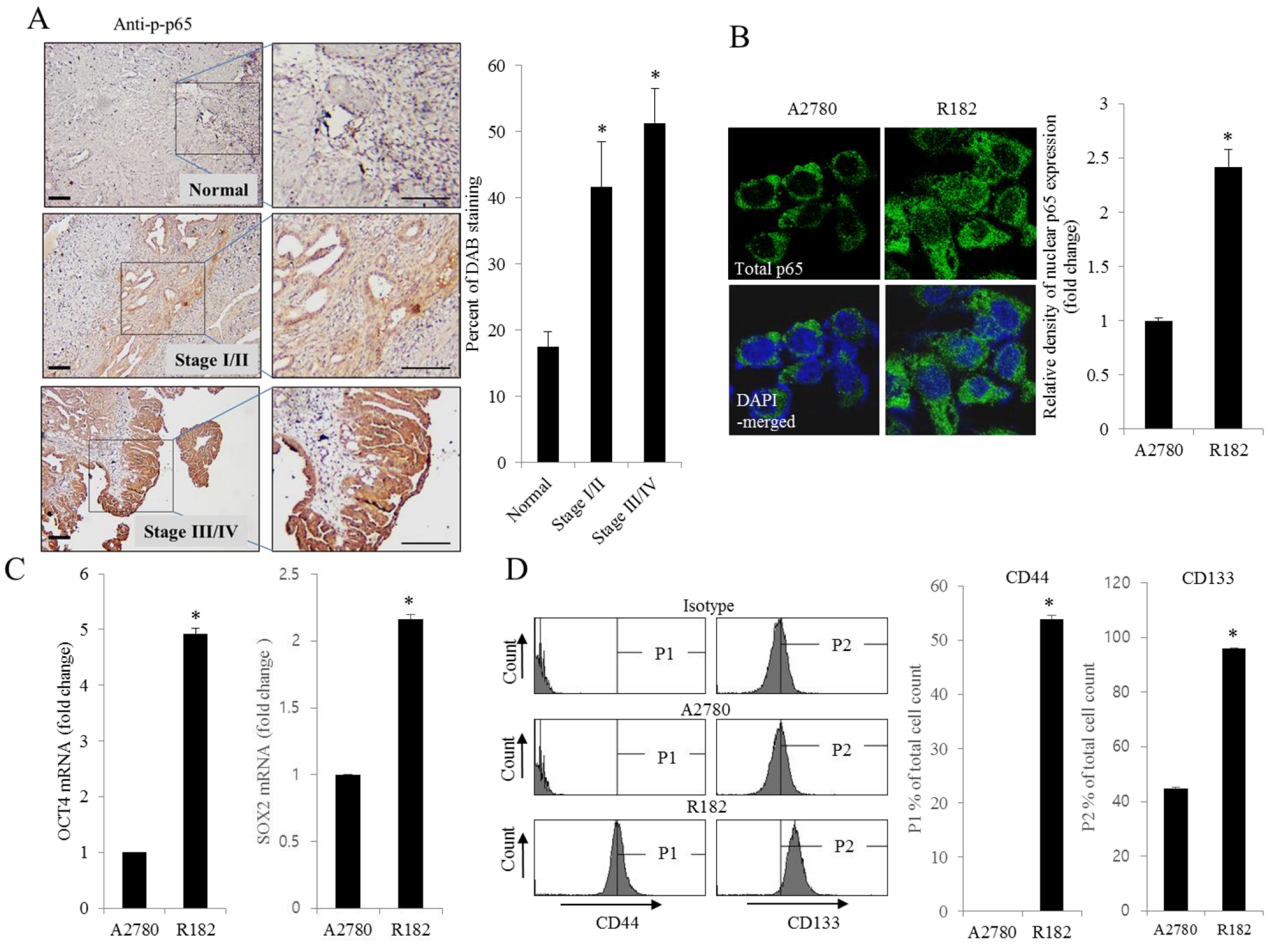
Histological and molecular phenotype of human ovarian cancer **A.** Paraffin sections of human ovarian tissues from normal or cancer patients were stained with anti-p-p65 Ab by the immunoperoxidase method as described in the Materials and Methods section (original magnification ×100) (Black scale bar, 0.2 μm). The right panel shows the percentage of p-p65-positive cells measured using HistoQuest tissue analysis software. *A significant difference from the normal group (*p*< 0.05). **B.** A2780 cells and R182 cells were stained with an anti-p65 Ab and DAPI before being assessed by confocal microscopy (original magnification ×1800). The right panel shows the relative density of nuclear p65 expression in the cell. *A significant difference from levels in A2780 cells (*p*< 0.05). **C.** mRNA levels of OCT4 and SOX2 in A2780 cells and R182 cells were measured using reverse transcription real-time PCR. *A significant difference from levels in A2780 cells (*p*< 0.05). **D.** Cellular fluorescence from binding of anti-CD44-FITC Ab and anti-CD133-APC Ab was measured using flow cytometry analysis. *A significant difference from levels in A2780 cells (*p*< 0.05).

**Figure 2:**
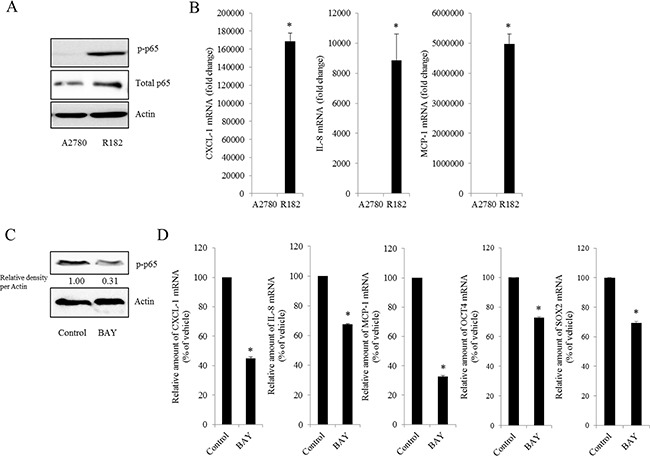
Effect of NF-κB activation on human ovarian cancer chemokines **A.** Cellular lysates of A2780 cells and R182 cells were subjected to western blot analysis. **B.** mRNA levels of A2780 and R182 cells were measured using reverse transcription real-time PCR. *A significant difference from levels in A2780 cells (*p*< 0.05). **C.** R182 cells were treated with control or 20 μM BAY 11-7082 for 4 h. Cellular lysates were subjected to western blot analysis. **D.** R182 cells were treated with the vehicle or 20 μM of BAY11-7082 for 4 h. mRNA levels were measured using reverse transcription real-time PCR. *A significant difference from the vehicle-treated group (*p*< 0.05).

As commented in the introduction, based on the morphologic and molecular genetic features, tissues of the type II tumors with high grade, aggressive and overwhelming p53 mutations were collected to perform the cell-based characterization of EOC. To determine the association between inflammatory signaling and the progression of human EOC, the phosphorylation status of p65 was measured in normal and advanced-cancer ovarian tissues from patients with stage I/II and III/IV ovarian cancer (*n* = 9). Like other cancers, the level of p65 phosphorylation in ovarian cancer samples was significantly higher than that in normal ovarian tissues (Figure 1A). To investigate the molecular mechanisms of p65 activation in EOC cells, MyD88-positive type I EOC cells (R182) were compared with a MyD88-negative human ovarian cancer cell line, A2780. Previous studies have shown that MyD88-activated R182 cells can produce pro-inflammatory and pro-tumorigenic cytokines, which can confer resistance to anti-cancer drugs [24, 25]. Expression of total p65 in R182 was relatively higher than that in A2780 and the nuclear translocation of p65 was also 2.5-fold higher (Figure 1B). Moreover, R182 cells showed enhanced levels of cancer stemness biomarkers such as OCT4, SOX2, CD44, and CD133, compared with the marginal expression of these factors in A2780 cells (Figure 1C and 1D). In addition to the elevation of total p65 levels, activated NF-κB and an enhanced expression of chemokines including CXCL-1, IL-8, and MCP-1 were observed in R182 cells, compared to their levels in A2780 cells (Figure 2A and 2B). Blocking of persistent NF-κB signals in R182 cells using BAY11-7082, a specific IKK inhibitor, significantly decreased p65 phosphorylation (Figure 2C) and subsequent chemokine expression (Figure 2D). However, retardation of IL-8 expression by IKK inhibition was only partial, implicating the presence of alternate or compensatory pro-inflammatory signals in addition to the well-known NF-κB-linked cascade.

### Expression of NAG-1 involves NF-κB activation, chemokine production, and cancer stemness

**Figure 3:**
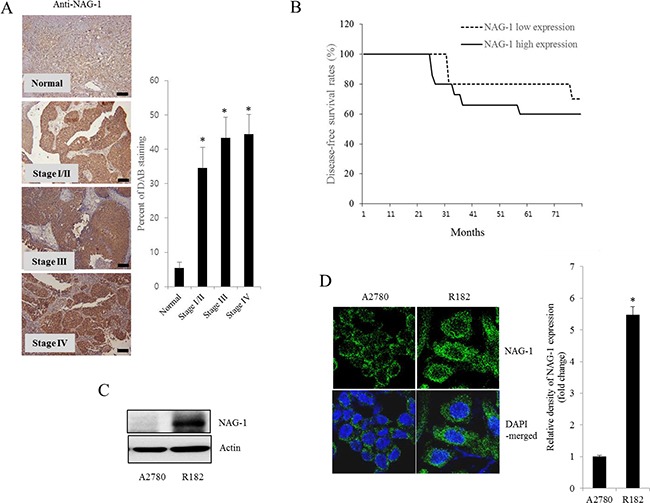
NAG-1 expression in human ovarian cancer and type I EOC cells **A.** Paraffin sections of human ovarian tissues from normal or cancer patients were stained with anti-NAG-1 Ab by the immunoperoxidase method as described in the Materials and Methods section (original magnification ×100) (Black scale bar, 0.2 μm). The right panel shows the percentage of cells stained for NAG-1 DAB measured using HistoQuest tissue analysis software. *A significant difference from the normal group (*p*< 0.05). **B.** Kaplan-Meier survival analysis of disease-free survival in ovarian cancer patients comparing the low NAG-1 expression group (n=10) and the high NAG-1 expression group (n=15). **C.** Cellular lysates of A2780 cells and R182 cells were subjected to western blot analysis. **D.** A2780 cells and R182 cells were stained with an anti-NAG-1 Ab and DAPI before being assessed by confocal microscopy (original magnification ×1800). The right panel shows the relative quantitative values of NAG-1 expression. *A significant difference from A2780 (*p*< 0.05).

**Figure 4:**
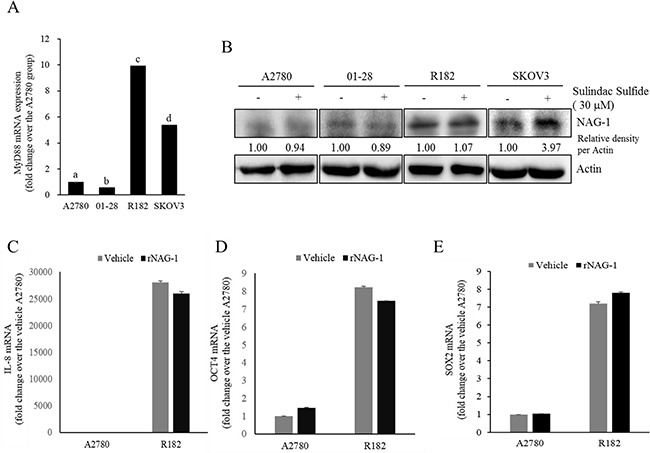
Effects of sulindac sulfide or recombinant NAG-1 (rNAG-1) on NAG-1-linked signals **A.** MyD88 mRNA levels in human EOC cell lines (A2780, 01-28, R182, or SKOV3) were measured using reverse transcription real-time PCR. Different letters represent significant difference between two groups (*p* < 0.05). **B.** A2780, 01-28, R182 or SKOV3 cells were treated with vehicle or 30 μM sulindac sulfide (S.S) for 48 h. Cellular lysates were subjected to Western blotting analysis. (C-E) A2780 and R182 cells were treated with vehicle or 10 ng/ml rNAG-1 for 24 h. Cellular mRNA levels were measured using reverse transcription real-time PCR.

**Figure 5:**
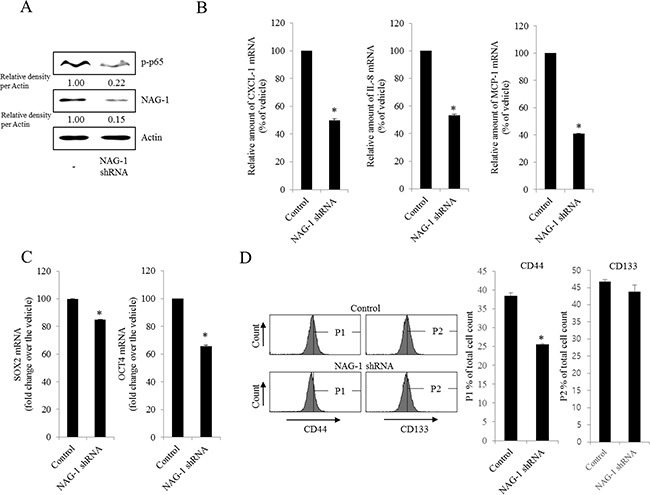
Involvement of NAG-1 expression in NF-κB-mediated inflammatory responses and stemness in R182 cells R182 cells expressing the control vector or NAG-1 shRNA plasmid were compared. **A.** Cellular lysates were subjected to western blot analysis. **B.** and **C.** mRNA levels were analyzed by reverse transcription real-time PCR. *A significant difference from the control (*p*< 0.05). **D.** Cellular fluorescence from the binding of anti-CD44-FITC Ab and anti-CD133-APC Ab was measured using flow cytometry analysis. *A significant difference from the control (p < 0.05).

**Figure 6:**
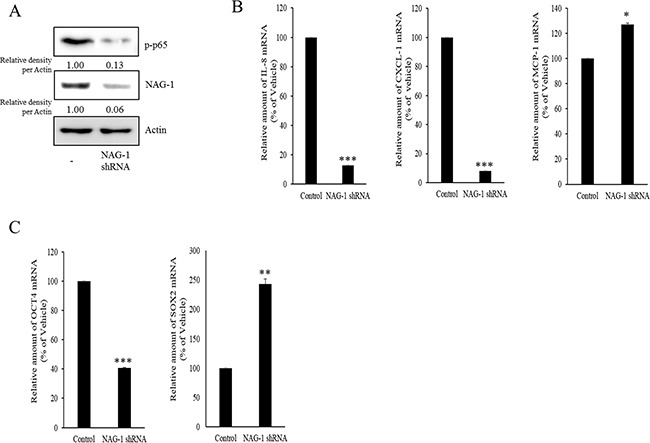
Involvement of NAG-1 expression in NF-κB-medicated inflammatory responses and stemness in SKOV3 cells **A.** Control or NAG-1 shRNA vector-transfected SKOV3 cells were subjected to Western blot analysis. **B.** and **C.** mRNA levels of control or NAG-1 shRNA vector-transfected SKOV3 cells was measured using reverse transcription real-time PCR. A significant difference from the control (*, *p*< 0.05; **, *p*< 0.01; or ***, *p*< 0.001).

As another potent marker of EOC progression, NAG-1 protein abundance was assessed in the present study. NAG-1 expression in advanced ovarian cancer tissues was markedly higher than that in normal samples (Figure 3A). Moreover, according to the survival analysis, the lower NAG-1 expression group had more survival chances than the higher NAG-1 expression group, indicating that NAG-1 expression was a potent biomarker of poor prognosis in patients with ovarian cancer (Figure 3B). Functionally, our recent studies have suggested that NAG-1 expression in cancer cells plays a pivotal role in maintaining a prolonged activation of inflammatory responses in the intestinal mucosal microenvironment [55, 57]. To determine the links between NAG-1 expression and NF-κB activation in the progress of ovarian cancer, the expression levels of NAG-1 protein were also measured in both R182 and A2780 cells. In agreement with our previous observations in human cancer tissues, MyD88-positive EOC R182 cells showed relatively enhanced levels of NAG-1 protein, compared with the levels of NAG-1 in MyD88-negative A2780 cells (Figure 3C and 3D). Based on the MyD88 expression levels, four EOC cell lines were classified into MyD88-high cells (R182 and SKOV3), and MyD88-low cells (A2780 and 01-28) (Figure 4A). Since NAG-1 has been known to be upregulated by NSAID treatment in epithelial cancer cells, we assessed the inductive actions of sulindac sulfide, a representative NAG-1-inducing NSAID, in the present EOC cell culture model. These cells were also compared for induction of NAG-1 by sulindac sulfide (Figure 4B). Among the EOC cell lines, only the SKOV3 cells showed prominent induction of NAG-1 in response to sulindac sulfide. Moreover, functionally active NAG-1 protein was assessed for its actions on cancer chemokines and stemness biomarkers in the EOC cell lines. However, treatment with recombinant mature NAG-1 had marginal effects on expressions of IL-8, OCT4, and SOX2 in A2780 and R182 cells (Figure 4C-4E). In contrast, suppression of NAG-1 expression using its shRNA led to a dramatic inhibition of p65 phosphorylation in R182 cells (Figure 5A). Additionally, NF-κB-mediated induction of chemokines such as CXCL-1, IL-8, and MCP-1 was significantly decreased by the genetic ablation of NAG-1 (Figure 5B). Moreover, the genetic ablation of NAG-1 suppressed the expression of cancer stemness biomarkers including SOX2, OCT4, and CD44 (Figure 5C and 5D). We also evaluated effects of NAG-1 suppression on SKOV3 cells. The genetic knockdown of NAG-1 also suppressed p65 phosphorylation (Figure 6A). Moreover, ovarian cancer chemokines such as IL-8 and CXCL-1 and stemness marker OCT4 were suppressed by NAG-1 in SKOV3 cells (Figure 6B and 6C), indicating that NAG-1 expression positively regulates not only pro-inflammatory signals but also cancer stemness biomarkers in MyD88-positive type I EOC cells.

### NAG-1 triggers NF-κB-dependent TGFR-linked and NF-κB-independent EGFR-linked signaling cascades

**Figure 7:**
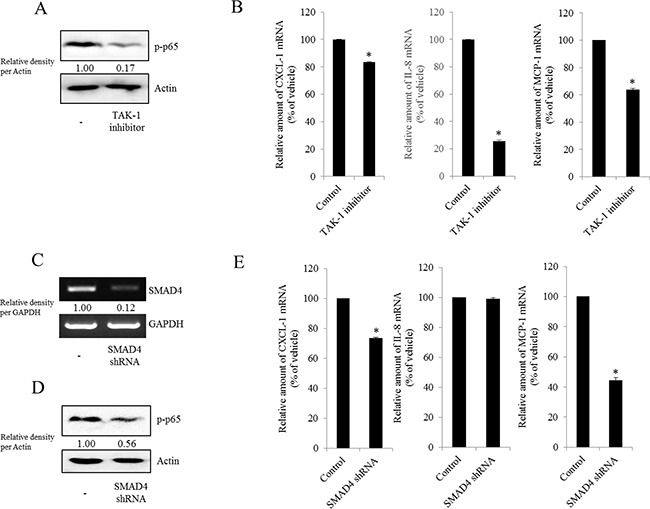
Involvement of TAK-1 and SMAD4 expression in NF-κB-mediated inflammatory responses in type I EOC cells R182 cells were treated with 2 μM of the TAK-1 inhibitor 5Z-7-oxozeaenol for 4 h. **A.** Cellular lysates were subjected to western blot analysis. **B.** mRNA levels were analyzed by reverse transcription real-time PCR. *A significant difference from the control (*p*< 0.05). (C–E) R182 cells expressing the control vector or SMAD4 shRNA plasmid were compared. **C.** SMAD4 expression were analyzed using RT-PCR. **D.** Cellular lysates were subjected to western blot analysis. **E.** mRNA levels were analyzed by reverse transcription real-time PCR. *A significant difference from the control (*p*< 0.05).

NAG-1 can bind to and activate TGF-β receptor II (TGFR-II), which can trigger both SMAD- and non-SMAD-linked signals. Whereas the SMAD signaling axis is known as the canonical pathway, SMAD-independent TGF-β-activated kinase-1 (TAK-1) also can mediate the downstream responses of TGFR-II-linked signals and affect other signal transduction pathways such as the MyD88-NF-κB pathway [58, 59]. Based on these findings, we performed experiments to address whether inhibiting TAK-1-linked signals could modulate NF-κB activation and subsequent cytokine expression in MyD88-positive EOC cells. The phosphorylation of NF-κB and subsequent chemokine expression were significantly suppressed by TAK-1 inhibition (Figure 7A and 7B), suggesting that TAK-1 is positively involved in NAG-1-mediated NF-κB activation and chemokine production in MyD88-positive EOC cells. Moreover, we also assessed the effects of NAG-1 on SMAD-linked signals. Activated TGFR-II enhances the phosphorylation of SMAD2 and SMAD3, which then bind to SMAD4 and form the SMAD complex, which in turn translocates into the nucleus to activate the transcription of its target genes [60, 61]. When suppressing SMAD-linked signaling transduction using a SMAD4-specific shRNA, NF-κB activation was attenuated in MyD88-positive EOC cells (Figure 7C and 7D). In addition, the genetic ablation of SMAD4 down-regulated chemokine expression to some extent (Figure 7E), suggesting that the SMAD-linked pathway contributes to NAG-1-regulated pro-inflammatory signals in MyD88-positive EOC cells.

**Figure 8:**
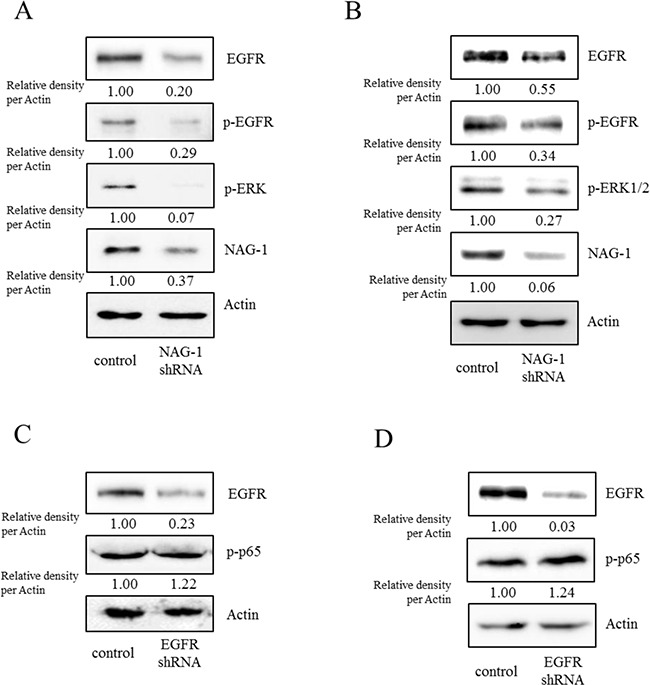
Involvement of NAG-1-mediated EGFR activation in inflammatory signals in type I EOC cells R182 **A, C.** and SKOV3 **B, D.** cells expressing the control vector or NAG-1 shRNA plasmid were compared. Cellular lysates were subjected to western blot analysis.

**Figure 9:**
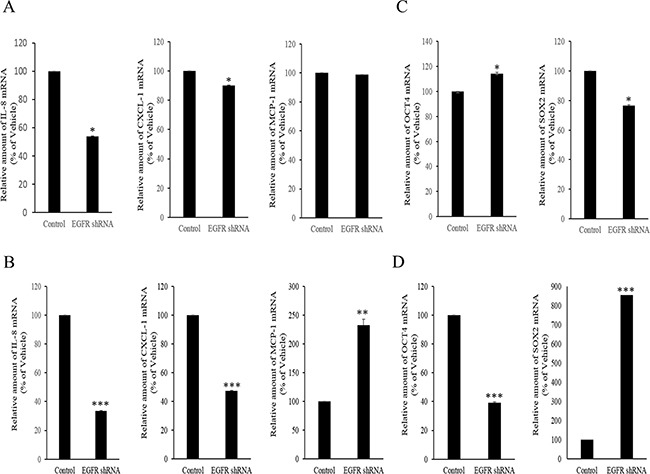
Involvement of NAG-1-mediated EGFR activation in inflammatory responses in type I EOC cells R182 **A, C.** and SKOV3 **B, D.** cells expressing the control vector or EGFR shRNA plasmid were compared. Cellular mRNA levels were analyzed by reverse transcription real-time PCR. A significant difference from the control (*, *p*< 0.05; **, *p*< 0.01; or ***, *p*< 0.001).

However, the expression of some chemokines was partially down-regulated by the inhibition of either the SMAD- or TAK-1-linked pathways, which indicates the presence of other signals for NAG-1-mediated cytokine induction. In addition to TGFR-II, epidermal growth factor receptor (EGFR) signaling has been known to be affected by members of the TGF-β superfamily [62–64]. We thus assessed the effects of NAG-1 on EGFR signaling cascades in type I EOC cells. Suppression of NAG-1 expression using its shRNA led to a significant inhibition of EGFR expression and its downstream signaling mediator ERK1/2 (Figure 8A and 8B), indicating the positive regulation of cancer EGFR-linked signals by NAG-1. However, the activated EGFR was not involved in NF-κB activation in MyD88-positive ovarian cancer cells (Figure 8C and 8D). In contrast, EGFR contributed to an enhanced production of cancer chemokines such as IL-8 and CXCL-1 in ovarian cancer cells (Figure 9A and 9B). However, expression of MCP-1 was not significantly reduced by EGFR suppression in R182 cells and even elevated in SKOV3 cells. Moreover, actions of EGFR in regulation of cancer stemness markers including OCT4 and SOX2 were not consistent in EOC cells (Figure 9C and 9D). Taken the consistent results, the present results demonstrated that the activation of EGFR was positively associated with the induction of cancer chemokines (IL-8 and CXCL-1) via NF-κB-independent pathways in type I EOC cells.

### NAG-1-mediated NF-κB activation increases chemoresistance to paclitaxel in R182 cells

**Figure 10:**
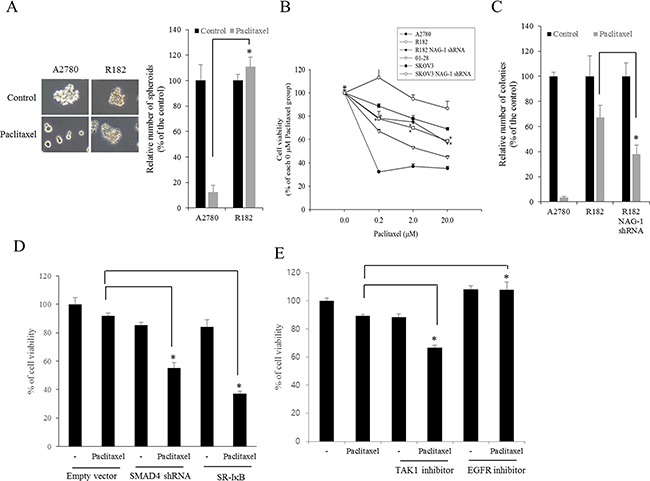
Involvement of NAG-1-mediated signals in drug resistance in ovarian cancer cells **A.** A2780 and R182 cells were treated with 1 μM paclitaxel for 4 days and the spheroids were counted. *A significant difference from the paclitaxel-treated A2780 cells (p < 0.05). **B.** The viability of EOC cells (A2780, 01-28, R182, or SKOV3 cells) expressing vector or NAG-1 shRNA plasmid were compared. Cells were treated with each dose of paclitaxel for 48 h, and cell viability was measured using MTT assay. *A significant difference from the each vehicle-treated group (*p*< 0.05). **C.** Colonies of EOC cells (A2780 or R182 cells) expressing vector or NAG-1 shRNA plasmid were treated with 1 μM paclitaxel for 48 h and then incubated for 4 days. The number of colonies were counted. *A significant difference from the paclitaxel-treated R182 cells (p < 0.05). **D.** R182 cells expressing the control vector, SMAD4 shRNA plasmid, or SR-IκB expression plasmid were compared. Each cells were treated with vehicle or 2 μM paclitaxel for 48 h, and cell viability was measured using MTT assay. *A significant difference from only the paclitaxel-treated group (p < 0.05). **E.** R182 cells were pre-exposed to control, 2 μM 5Z-7-oxozeaenol (TAK-1 inhibitor) or 1 μM AR1478 (EGFR inhibitor) and treated with vehicle or 2 μM paclitaxel for 48 h. *A significant difference from only the paclitaxel-treated group (*p*< 0.05).

Cancer NF-κB and chemokines are crucial in mediating resistance to chemotherapy. Therefore, effects of chemotherapeutics on cancer spheroid formation were compared between MyD88-positive EOC cells (R182) and MyD88-deificient EOC cells (A2780). Paclitaxel, a taxane drug used as a component of standard chemotherapy combinations against EOC, significantly inhibited spheroid formation of A2780 cells whereas it marginally affected the formation of R182 spheroid (Figure 10A). Moreover, we addressed whether NAG-1 was also involved in modulating chemoresistance in MyD88-positive type I EOC cells. We assessed the effects of NAG-1 suppression on EOC cell viability in response to paclitaxel. As expected, MyD88-high R182 and SKOV3 cells were more resistant to paclitaxel-induced cytotoxicity than MyD88-low A2780 and 01-28 cells (Figure 10B). However, the genetic ablation of NAG-1 significantly increased the paclitaxel-induced cytotoxicity in R182 and SKOV3 cells. Likewise, colonies of R182 cells were more resistant to paclitaxel than those of A2780 cells, but suppression of NAG-1 using its shRNA significantly decreased the number of R182 colonies surviving from paclitaxel-induced cytotoxicity, confirming the involvement of NAG-1 in mediating the resistance to paclitaxel in the type I EOC cells (Figure 10C). In terms of signaling transduction of chemosensitivity, the suppression of NF-κB signaling as a key pathway of drug resistance using super-repressor mutant IκB (SR-IκB) also significantly enhanced the chemosensitivity of R182, the type I EOC cells (Figure 10D). Moreover, SMAD signals and the non-SMAD-linked mediator TAK-1, which activate NF-κB signaling, were also positively involved in the EOC cell resistance to paclitaxel (Figure 10D and 10E). In contrast, the inhibition of EGFR, which transmits signals via an NF-κB-independent pathway, did not alter the viability of R182 cells in response to paclitaxel (Figure 10E), indicating that chemoresistance depends on stimulation by NF-κB-activating NAG-1 in type I EOC cells.

**Figure 11:**
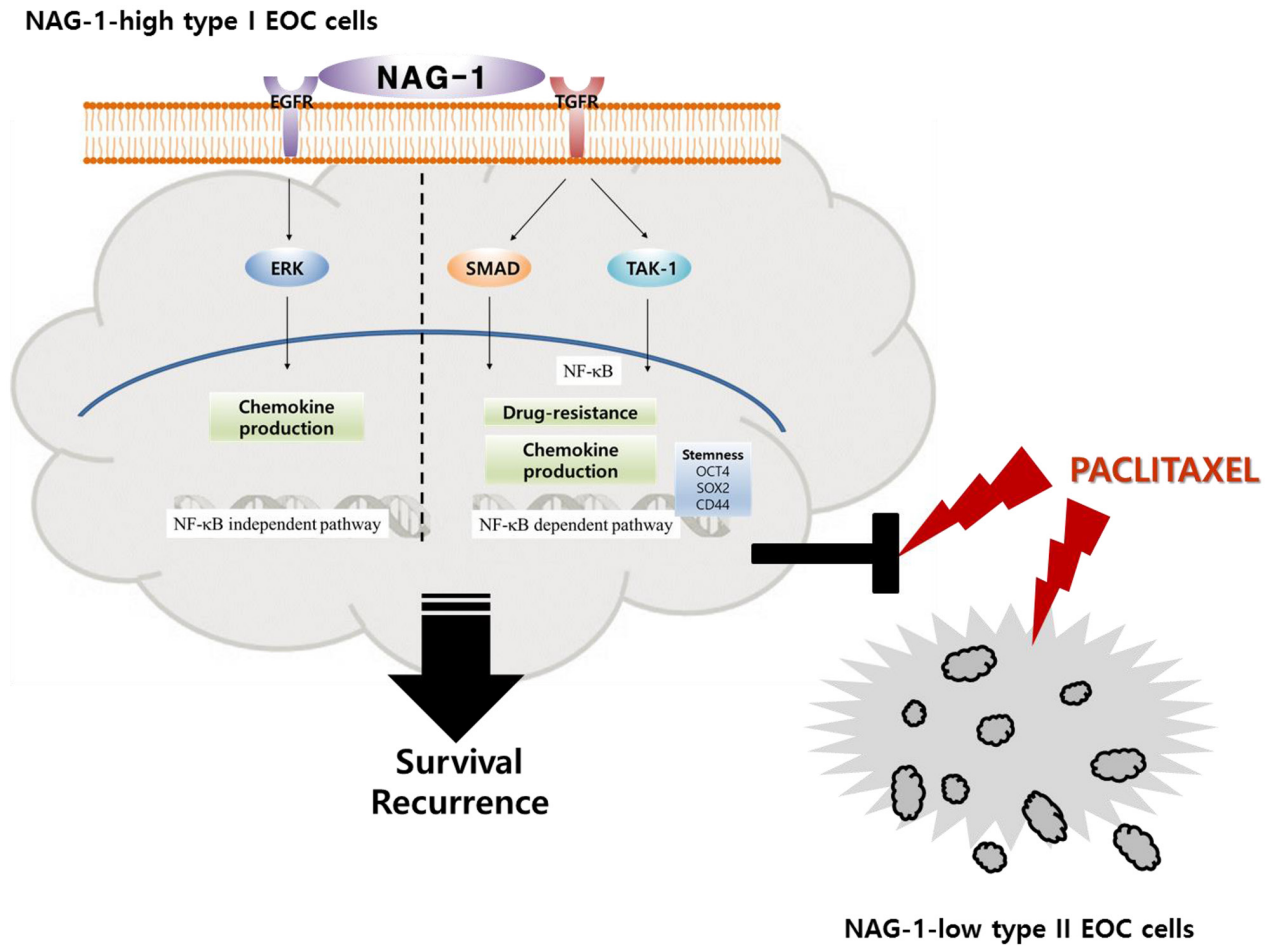
A putative scheme for the mechanism of NAG-1-mediated chemokine production and chemoresistance in type I EOC cells Abnormal expression of NAG-1 triggers NF-κB activation via TGFR-mediated signaling cascades including SMAD and TAK-1, which play critical roles in chemokine production and chemoresistance against paclitaxel treatment in MyD88-positive type I EOC cells. Moreover, NAG-1 and NF-κB showed partial effects on increasing the expression of ovarian stemness markers, such as OCT4, SOX2, and CD44. In addition, NAG-1-mediated EGFR-ERK signaling activation was majorly involved in NF-κB-independent chemokine production. However, MyD88-deficient EOC type II cells were sensitive to paclitaxel treatment, compared to the responses in MyD88-positive type I EOC cells, due to deficiency of NAG-1 expression and NF-κB activation.

Taken together, our results showed that NAG-1 was a crucial activator of pro-inflammatory signaling responses in type I EOC cells, partly due to the activation of NF-κB-dependent SMAD and TAK-1 signaling and NF-κB-independent EGFR signaling. Moreover, cancer stemness biomarkers and paclitaxel resistance in type I EOC cells were positively regulated by NAG-1 protein, which appears to act as a mediator of EOC recurrence and chemoresistance under chronic inflammatory stimulation (Figure 11).

## DISCUSSION

Recurrence and chemoresistance are the major obstacles to successful EOC management. Chemotherapy eliminates the bulk of the highly proliferative tumor cells but leaves a core of EOC stem cells with a high capacity for repair and renewal [27]. In the present report, evidence was demonstrated for the involvement of NAG-1 in pro-inflammatory activation, cancer stemness, and chemoresistance in human type 1 EOC cells. Mechanistically, NAG-1 mediated NF-κB activation through the SMAD4 or TAK-1-linked signaling pathways, which was critical for EOC drug resistance. Moreover, inflammatory cytokine production was induced in EOC cells not only by SMAD4-associated NF-κB activation, but also by a NF-κB-independent NAG-1-mediated EGFR signaling cascade. Persistent activation of NF-κB was explained by high NAG-1 expression in human ovarian cancer tissue samples and type I EOC cells (R182 cells) compared with its levels in normal tissues and type II EOC cells (A2780 cells). This is the first report on NAG-1 as a positive modulator of pro-inflammatory signals, cancer stemness, and chemo-resistance in human EOC. Elevated NF-κB activation maintains the production of cytokines and chemokines that are strongly associated with cancer progression, recurrence, and chemoresistance [11–14], and thus endogenous upstream stimulators in cancer cells need to be managed to control persistent NF-κB-linked events in EOC.

Various types of receptors including toll-like receptors (TLRs) and growth factor receptors have been demonstrated to induce an EOC microenvironment and cancer stemness characterized by raised levels of pro-inflammatory molecules including cytokines, resulting from NF-κB activation [65–67]. Consistently, the present study suggested that NAG-1 modulates chronic NF-κB phosphorylation via TGFR-II-linked signals that can activate SMAD proteins, which in turn can interact with NF-κB proteins, such as p52, leading to the transcription of pro-inflammatory genes [60]. Although ovarian cancer cells are known to lose their responsiveness to inhibitory growth signals exerted by TGF-β by mechanisms including the down-regulation of TGF-β receptors, TGF-β may indirectly affect ovarian tumor growth by modulating the secretion of stroma-specific mediators in the tumor microenvironment [68–70]. Moreover, previous studies from our group have suggested that NAG-1 modulates chronic NF-κB phosphorylation via the activation of TGF-β-activated kinase-1 (TAK-1) as one of the major kinases activated by TGF-β [55, 57]. In particular, TAK-1 is thought to be a signaling kinase critical for MyD88-NF-κB activation via phosphorylation of the inhibitor-κB kinase (IKK) complex, which leads to the transcription of pro-inflammatory genes [57, 59]. Ser412 phosphorylation of IKK by TAK-1 leads to activating NF-κB signaling, which promotes the aggressiveness of ovarian cancer cells [71]. In response to cancer chemotherapy, the inhibition of TAK-1 induces ROS and decreases the chemoresistance of cancer cells by promoting apoptosis [72–74]. In addition to its effects on cancer chemotherapy, TAK-1 is significantly associated with high-grade and metastatic ovarian cancers, and the blocking of TAK-1 remarkably impairs tumor growth and metastasis in ovarian cancer. Although NF-κB activation and subsequent chemokine expression was closely associated with NAG-1-activated signals in EOC cells, IL-8 was not significantly reduced by the blocking of SMAD4 expression (Figure 7). This result might be due to the partial suppression of NF-κB activation by SMAD4 genetic ablation and thus NF-κB-independent inflammatory signals, including EGFR signaling cascades, may have compensatory effects on IL-8 induction. In addition to SMAD-linked gene regulation, TAK-1 also contributed to NAG-1-activated p65 phosphorylation and chemokine production in type I EOC cells (Figure 7). As suggested in the present study, TGF-β receptor II (TGFR-II)-linked TAK-1 is expected to be another promising target for interventions to reduce EOC progression and recurrence. Moreover, as a major inducer of NF-κB activation, the mechanism of the TLR-MyD88 signaling pathways is well established. Previous studies have demonstrated that the activation of TLR4-MyD88 signaling favors tumor growth and chemoresistance in ovarian cancer [24]. However, the genetic ablation of NAG-1 did not alter the levels of TLR2 and TLR4 mRNA expression (data not shown), indicating that NAG-1 is likely to be another positive regulator of NF-κB activation, bypassing TLR2/4 in EOC cells. In the present study, NAG-1-activated NF-κB signal was closely associated with expression of cancer stemness markers. Likewise, IKK alpha activity is required for self-renewal of ErbB2/Her2-transformed mammary tumor-initiating cells [75] and the canonical NF-κB pathway is active in normal luminal progenitor cells before transformation [76]. In EOC cell models, Twist-1, a transcription factor involved in the process of differentiation, is highly expressed in Type II but not expressed in Type I EOC stem cells [77]. Although Twist-1 is a key modulator of cell differentiation and organ development, it can downregulate the IKK beta/ NF-κB pathways by enhancing the expression of miR199a in Type II EOC cells [77]. Therefore, through regulation of the convergent master switcher such as Twist-1 between differentiation and pro-inflammatory signal, the cancer progenitor cells can be maintained in the undifferentiated states. Further investigations are thus warranted to address the master modulator in NAG-1-activated pro-inflammatory signals and cancer stemness in the complex EOC niche.

Accumulating evidence has indicated that the up-regulation of NAG-1 expression is associated with an increased risk of various human cancers [42–48]. In addition to the effects of NAG-1 on pro-inflammatory signals and drug resistance, elevated levels of serum NAG-1 in patients are directly linked to the progression of tumors to metastatic stages [44, 51, 53] by its modulation of cancer cell survival and motility in the extracellular matrix and circulation [50, 54, 55]. Since NF-κB is generally less associated with the metastatic activities of malignant tumors, other targets of NAG-1, including AKT, ERK1/2 [54, 78], and FAK-RhoA GTPase [79] may also contribute to distant metastasis via enhanced locomotive activity and resistance to anoikis stress during migration in the circulation [80]. Especially, the phosphorylation of EGFR can cause ERK1/2 MAPK signaling activation, which contributes to cell proliferation, survival, and metastasis, as well as inflammation [81, 82]. In agreement with these reports, the activation of EGFR signaling led to chemokine production in a partially NF-κB independent manner (Figure 8 and Figure 9). However, EGFR activation was not positively associated with cancer cell viability in response to paclitaxel in R182 cells (Figure 10E). In the present study, NAG-1 affected the expression of EGFR and subsequently suppression of NAG-1 expression downregulated EGFR-linked signals such as ERK1/2 MAPK. In terms of transcription, EGFR promoter contains binding sites of p53, a key target protein of NAG-1 signaling pathway [83]. However, EGFR promoter activity can be enhanced by Sp1 in p53-independent pathways [84]. Smad as a key transcription factor of TGFβ superfamily-linked signal is the potent binding partner of Sp1 and their binding modulates TGFβ-induced target gene expression [85, 86], which may account for NAG-1-induced EGFR expression in the present model. As another candidate metastasis-associated signaling factor in association with NAG-1 expression, activating transcription factor 3 (ATF3) can enhance tumor progression by inducing genes involved in tumor metastasis, which is advantageous for malignant cancer cells [87]. Moreover, ATF3 directly affects NAG-1 transcription [88], and we also observed a high expression of ATF-3 in R182 cells by western blotting (data not shown), suggesting that ATF3 might modulate NAG-1-linked cancer progression and drug resistance. In the present study, effects of NAG-1 on EOC cell metastasis were not evaluated, and thus additional studies on the influences of enhanced NAG-1 expression on EOC cell metastasis are warranted to elucidate the broad spectrum of cancer events influenced by NAG-1.

In summary, our results demonstrated that the advanced stage of EOC was closely associated with elevated NAG-1 expression, which also contributed to prolonged pro-inflammatory signals. These NAG-1-associated cellular signals also mediated increases in resistance to cancer chemotherapy, cancer stemness, and cancer cell survival. In order to improve and advance the management of EOC, it is critical to expand our understanding of the biology of type I EOC stem-like cells and their dynamics in the complex tumor population. The identification of ovarian cancer stem cells and their crucial modulator NAG-1 may contribute to the early detection of EOC as well as the treatment and prevention of recurrence and chemoresistance in EOC patients. The present findings implicate high NAG-1 production as a potential diagnostic marker for EOC progression and chemoresistance, providing additional understanding of therapeutic strategies in inflammation-associated tumorigenesis. Extensive studies are also needed to address the clinical effect of targeting NAG-1 in EOC patients with drug resistance associated with features of EOC stemness in the inflammatory tumor niche.

## MATERIALS AND METHODS

### Cell culture and reagents

Established type I human EOC R182 cells and 01-28 (MyD88-negative) primary EOC cells were kindly provided by Prof. Gil Mor (Yale University School of Medicine, CT, USA). A2780 type II human EOC cells and SKOV3 (MyD88-positive) cells were purchased from American Type Culture Collection (Manassas, VA, USA). Cells were maintained in RPMI 1640 medium supplemented with 20% (v/v) heat-inactivated fetal bovine serum (FBS), 50 U/ml penicillin, and 50 mg/ml streptomycin (all from Welgene, Daegu, South Korea) in a 5% CO_2_ humidified incubator at 37°C. Cell numbers were counted by trypan blue (Sigma-Aldrich, St. Louis, MO, USA) dye exclusion assay using a hemocytometer. Sulindac sulfide (≥98% HPLC) was purchased from Sigma-Aldrich. Recombinant NAG-1 protein was purchased from Peprotech (Rocky Hill, NJ, USA).

### Western immunoblot analysis

Expression levels of protein samples were assessed by western immunoblot analysis using rabbit polyclonal anti-human actin Ab, goat polyclonal anti-human NAG-1 Ab, mouse monoclonal anti-human p-ERK1/2 Ab, rabbit polyclonal anti-human p65 Ab (Santa Cruz Biotechnology, Santa Cruz, CA, USA), rabbit polyclonal anti-human phosphorylated (p)-p65 Ab (Cell Signaling Technology, Beverly, MA, USA), rabbit monoclonal anti-human p-EGFR Ab (Millipore, Billerica, MA, USA), rabbit monoclonal anti-human EGFR Ab (Epitomics Burlingame, CA, USA), mouse monoclonal FITC-conjugated anti-human CD44 Ab (BD Biosciences, Franklin Lakes, NJ, USA), mouse monoclonal APC-conjugated anti-human CD133 Ab (Miltenyi Biotec, Bergisch Gladbach, Germany), and anti-rabbit secondary Ab (Enzo Life Science, Plymouth Meeting, PA, USA). Cells were washed with ice-cold phosphate buffer, lysed in boiling lysis buffer (1% [w/v] SDS, 1 mM sodium orthovanadate, and 10 mM Tris [pH 7.4]), and sonicated for 30 seconds. Protein lysates were quantified using a bicinchoninic acid protein assay kit (Welgene). Aliquots containing 50 μg of protein were separated by electrophoresis on polyacrylamide mini gels (Bio-Rad, Hercules, CA, USA). Proteins were transferred onto polyvinylidene fluoride membranes (Pall Corporation, NY, USA) and the blots were blocked for 1 h with 5% (w/v) skim milk in Tris-buffered saline plus 0.1% (v/v) Tween^®^ 20 (TBST) and then probed with primary Abs overnight at 4°C. After washing 3 times with TBST, blots were incubated with horseradish-conjugated secondary Ab for 2 h and then washed again 3 times with TBST. Ab binding proteins were detected using an enhanced chemiluminescence substrate (ELPIS Biotech, Taejon, South Korea).

### Immunohistological assessment of clinical samples

Ovarian cancer tissue samples were collected from ovarian cancer patients. All patients signed consent forms and the use of patient samples was approved by the institutional review board of Pusan National University Hospital (PNUH #1007-006-001, PNUH #1007-007-001). Formalin-fixed paraffin-embedded tissues from human ovary were cut, deparaffinized, and rehydrated. The tissue sections were heated in 10 mM sodium acetate (pH 9.0) for 5 min at 121°C for antigen retrieval. To remove endogenous peroxidase, tissues were bathed in a 3% (v/v) H_2_O_2_-PBS solution for 15 min at room temperature in the dark. After samples were washed with Tris-HCl–Tween (0.5%, v/v), and blocked with 3% (w/v) bovine serum albumin (BSA) in PBS for 1 h, they were incubated with the primary antibodies (1:200 dilution) overnight at 4°C. After washing 3 times with PBS, samples were incubated with the horseradish peroxidase-conjugated secondary antibody for 2 h at room temperature and then washed with PBS 3 times. The bound antibodies were identified using freshly prepared substrate buffer (0.05% [w/v] diaminobenzidine (DAB; Sigma-Aldrich) and 0.015% [v/v] H_2_O_2_ in PBS) for 2 min. After a final wash in PBS and distilled water, the sections were counterstained with 20% (v/v) hematoxylin (Santa Cruz Biotechnology) solution for 1 min and dehydrated. Sections were examined at various magnifications using an Axio Imager microscope (Carl Zeiss MicroImaging, GmbH, Oberkochen, Germany). Images of normal tissue and lesions were captured and processed using Motic^®^ Images Plus 2.0 following image acquisition. Quantification of the relative intensity of DAB staining was performed using the Histo-Quest software 4.0 (TissueGnostics, Vienna, Austria).

### Reverse transcription and conventional or real-time PCR

Total RNA was extracted using RiboEX™ reagent (GeneAll Biotech, Seoul, South Korea) according to the manufacturer's instructions. RNA (4 μg) from each sample was reverse transcribed into cDNA using a TOPscript™ RT DryMIX kit (Enzynomics, Seoul, South Korea). The amplification step was performed using n-Taq DNA polymerase (Enzynomics) in a MyCycler™ Thermal Cycler (Bio-Rad Laboratories) using the following parameters: initial denaturation at 95°C for 2 min followed by cycles of denaturation at 95°C for 30 seconds, annealing at 58°C for 30 seconds, and elongation at 72°C for 30 seconds. The 5′ forward and 3′ reverse-complement PCR primers for amplifying each gene were as follows: human IL-8 (5′-ATG ACT TCC AAG CTG GCC GTG GCT-3′ and 5′-TCT CAG CCC TCT TCA AAA ACT TCT C-3′), human chemokine (C-X-C motif) ligand 1 (CXCL-1) (5′-CTG CTC CTG CTC CTG GTA C-3′ and 5′-TGG ATT TGT CAC TGT TCA GCA-3′), human MCP-1 (5′-TCT GTG CCT GCT GCT CATAG-3′ and 5′-TGG AAT CCT GAA CCC ACT TC-3′), human OCT4 (5′-GCA AAG CAG AAA CCC TCG TG-3′ and 5′-AGC CTG GGG TAC CAA AAT GG-3′), human SOX2 (5′-GAC TTG ACC ACC GAA CCC AT-3′ and 5′-AAC CAG CGC ATG GAC AGT TA-3′), human SMAD4 (5′-CCA TCC AGC ATC CAC CAA GT-3′ and 5′-AGG CTG GAA TGC AAG CTC AT-3′), human MyD88 (5′-TTG AGG AGG ATT GCC AAA AG-3′ and 5′-GCG GTC AGA CAC ACA CAA CT-3′), and human GAPDH (5′- TCA ACG GAT TTG GTC GTA TT-3′ and 5′-CTG TGG TCA TGA GTC CTT CC-3′). The PCR products were electrophoretically separated on a 1% (w/v) agarose gel and visualized by ethidium bromide staining. For real-time PCR, SYBR^®^ Green was used as the fluorescent reporter dye to detect amplified cDNA. Real-time PCR was conducted using a Rotor Gene Q thermal cycler (Qiagen, Hilden, Germany) to subject the samples to initial denaturation at 95°C for 15 min and 40 cycles of denaturation at 95°C for 10 seconds, annealing at 60°C for 15 seconds, and elongation at 72°C for 30 seconds. Each experiment included 3 replicates to ensure statistical significance and each independent experimental set was repeated 2 or 3 times. The relative quantification of gene expression was performed using the comparative Ct method. The Ct value is defined as the point where a statistically significant increase in the fluorescence is observed. The number of PCR cycles (Ct) required for the SYBR^®^ Green fluorescence intensity to exceed a threshold level just above background was calculated for the test and reference reactions. In all experiments, GAPDH was used as the endogenous control. The results were analyzed by quantitation relative to the values for the control cells.

### Transient transfection and plasmids

R182 cells were transfected with combinations of plasmids using iN-fect™ transfection reagent (iNtRON, Seoul, South Korea) according to the manufacturers’ protocols. All transfection efficiencies were maintained around 50–60% and confirmed by expression of a pMX-green fluorescent protein vector. iN-fect transfection reagent was used to transfect vehicle or shNAG-1 transiently and cells were incubated for 48 h. EGFR shRNA targeted the sequence 5′-CTC TGG AGG AAA AGA AAG T-3′. SMAD4 shRNA was obtained from Addgene (Cambridge, MA, USA). SMAD4 shRNA targeted the sequence 5′-GGT GTG CAG TTG GAA TGT A -3′. An NAG-1 short hairpin RNA (shRNA) expression vector was kindly provided by Jong-Sik Kim (Andong National University, South Korea) and Seong-Joon Baek (University of Tennessee, TN, USA). NAG-1 shRNA targeted the sequence 5′-ACA TGC ACG CGC AGA TCA A-3′. Construction of the SR-IκBα-Flag expression vector has been described previously [89].

### Flow cytometry

Trypsinized cells (5 × 10^5^) were prepared in 1 ml of culture media. The cells were washed with PBS and immediately fixed using 4% (w/v) paraformaldehyde for 10 min. After washing 3 times with PBS, cells were permeabilized by adding permeabilization buffer (0.1 mM EDTA, 0.1% (v/v) Triton™ X100 in PBS) at RT for 20 min and then washed repeatedly with PBS. After blocking with 10% (v/v) FBS in PBS for 30 min, cells were incubated in buffer (10% [v/v] FBS in PBS) containing a 1:11 dilution of mouse monoclonal FITC-conjugated anti-human CD44 Ab (Becton Dickinson Biosciences, San Jose, CA, USA) and mouse monoclonal APC-conjugated anti-human CD133 Ab (Miltenyi Biotec, Bergisch Gladbach, Germany) primary Ab at 4°C for 30 min and washed thoroughly with PBS. Single-cell fluorescence was measured and analyzed using a FACSCanto™ II flow cytometer (Becton Dickinson Biosciences). Data from 10,000 cells were collected in list mode.

### Confocal microscopy

Cells were incubated in a glass-bottomed culture dish (SPL Life Sciences, Pocheon, South Korea). After treatment, cells were fixed in 4% (w/v) paraformaldehyde diluted in PBS, permeabilized with 0.2% (v/v) Triton™ X-100 and 0.3% (w/v) BSA in PBS for 10 min, blocked with 3% (w/v) BSA in PBS for 2 h, and incubated with primary Ab (1:200) in buffer (3% [w/v] BSA in PBS) at room temperature for 2 h. Next, the cells were repeatedly washed with PBS and incubated with Alexa Fluor 488 goat anti-rabbit IgG (H+L; Invitrogen) and FITC-anti-goat IgG for 2 h at room temperature, washed in PBS, and stained with 100 ng/ml DAPI (absorbance at 405 nm) in PBS for 10 min. Confocal images were obtained using an Olympus FV1000 confocal microscope (Olympus, Tokyo, Japan) and processed using FV10-ASW software (Olympus). The intensity of signals from 4 selected fields was measured using MultiGauge software (Fujifilm, Tokyo, Japan).

### Measurement of colony formation

Considering the different growth rates of two cell lines, five hundred A2780 cells or three thousands R182 cells were seeded onto the 100 mm cell culture dish. On the 6^th^ day after seeding, cell colonies were treated with 1 μM paclitaxel for 48 h and further incubated in the complete media without paclitaxel for 4 days. Colonies were washed with PBS and fixed with 100% methanol for 10 min at 37°C and survived colonies were stained with Giemsa for 30 min at room temperature. After washing with the distilled water 5 times, stained colonies were counted.

### Measurement of spheroid formation

Considering the different growth rates of two cell lines, 8 × 10^4^ A2780 cells or and 2.5 × 10^5^ R182 cells were seeded onto the Ultra-low attachment 6 well plate (Corning Incorporated Life Science, Lowell, MA, USA) in serum-free DMEM/F12 media (Welgene) supplemented with 20 ng/mL human recombinant epidermal growth factor (EGF; BD Biosciences), 20 ng/mL basic fibroblast growth factor (bFGF; Peprotech, Rocky Hill, NJ, USA), and 2% (v/v) B27 (Thermo Fisher scientific, Waltham, MA, USA). Cells were then treated with 1 μM paclitaxel for 4 days, and then spheroid formation of cells were measured under the microscope (Motic Electric Group Co Ltd, Xiamen, China).

### Measurement of cell viability

Epithelial ovarian cancer cell lines were seeded into 96-well plates at 5,000 cells per well and were incubated for 24 h. The medium was removed and fresh medium or paclitaxel was added in a final volume of 200 μl/well. The culture medium was removed after 48 h of incubation and 50 μl of PBS containing 1 mg/ml 3-(4,5-Dimethylthiazol-2-yl)-2,5-diphenyltetrazolium bromide (MTT) was added to each well and incubated for 4 h. The PBS containing MTT solution was carefully removed, then 200 μl of dimethyl sulfoxide was added to each well and incubated for 10 min at room temperature. A 96-well micro plate reader (Molecular Devices, Sunnyvale, CA, USA) was used to determine the absorbance at 540 nm.

### Statistical analysis

Data were analyzed using SigmaStat for Windows (Jandel Scientific, San Rafael, CA, USA). To compare data between 2 groups, a Student's t test was performed. To compare multiple groups, data were subjected to analysis of variance and pairwise comparisons were made by the Student-Newman-Keuls post hoc method.
